# Impacts of Perceived Discrimination During the COVID-19 Pandemic on Depression Among Older Chinese Immigrants in Aotearoa New Zealand

**DOI:** 10.1177/08982643251359150

**Published:** 2025-07-07

**Authors:** Polly Yeung, Christine Stephens, Gloria Gao, Rachel Huang

**Affiliations:** 1 School of Social Work, 6420 Massey University, Palmerston North, New Zealand; 2 School of Psychology, 6420 Massey University, Palmerston North, New Zealand; 3 CNSST Foundation, Auckland, New Zealand

**Keywords:** racism, health conditions, language barriers, COVID-19, depression

## Abstract

Racial discrimination against Chinese immigrants to various countries worldwide has risen sharply during the COVID-19 pandemic, but limited research exists regarding the pathways through which racial discrimination impacts older immigrants’ mental health. This study explored the relationship of perceived discrimination to depression among older Chinese immigrants living in Aotearoa New Zealand, through pathways of chronic health conditions, language barriers, and COVID-19 risks while taking into account the effects of anxiety and loneliness. Descriptive and regression analysis was conducted from a convenience sample of 1159 older Chinese immigrants aged between 55 and 80. While there was no significant direct effect of perceived discrimination to depression, the results showed a significant indirect effect of perceived discrimination, chronic illnesses, COVID-19 risks, and language barriers on depression, which was mediated by anxiety and loneliness. Ageing policies and interventions must address anti-racism to reduce the social and health inequalities faced by older ethnic people.

## Introduction

Older immigrants are at risk of poorer mental health. Older ethnic minority people are among the most excluded groups in society, due to experiences of negative acculturation, racist discrimination, and disadvantage that have accumulated over the life course (Boen et al., 2019; Choi, 2012; Stopforth, 2021; Wu et al., 2021). Those who immigrate at older ages (often through family reunion schemes to join their adult children) are particularly vulnerable to experiencing loneliness and social isolation due to prejudice, low financial security, and language barriers to navigating healthcare systems (Caidi et al., 2020). Older Chinese immigrants have been shown to have lower mental health compared to other older populations and experience more challenges in adaptation in a new country due to language barriers and migratory grief, which includes losses of cultural identity, sense of belonging, and social support networks (Bhatia et al., 2024; Park & Kim, 2013; Zhang et al., 2013).

In Aotearoa New Zealand (NZ), people aged 65 and older constitute 15% of the population, of which 6.3% identify as Asian (Statistics New Zealand, 2020). In line with the ageing of the general population, the NZ Chinese population is ageing rapidly with numbers of older people increasing from 9263 in 2006, to 23,625 in 2018; a 255% rise within 12 years (Statistics NZ, 2020). Since 1987, people from People’s Republic of China (hereafter China) have become the second-largest immigrant group in NZ (Immigration New Zealand, 2019a) due to the Immigration Act. The changes in immigration policies also saw late-life immigration among older people for the purpose of family reunification under the Immigration New Zealand’s Parent Category Family Reunion Scheme (Immigration New Zealand, 2019b). Ran and Liu (2021) noted that once adult immigrants settle in NZ, they usually hope to invite their parents to join them via the Family Reunification Category. Many older people have come to NZ to provide care for their adult children and grandchildren in host countries. However, a significant number of older immigrant parents had also been brought to NZ by their adult children sponsors who then left NZ for other countries, leaving their older parents ageing here alone (Pearson, 2014; Tan, 2013).

Ageing in a foreign land is a complicated journey. Chinese adults who immigrated in later-life face the same challenges to mental health identified in the international literature, including language difficulties, social adjustment, loneliness, and health care access (Montayne et al., 2019; Zhao et al., 2022). However, there has been very little research in NZ on mental health adjustment. The most recent study (Abbot et al., 2003) reported that these challenges, in addition to low emotional support, increased the risk of depression among older Chinese immigrants. Abbot and colleagues found elevated depressive symptoms among 25% of their city sample which was similar to the rate found by Liu et al. (1997) among a rural sample. These studies did not distinguish later-life immigrants from older adults who immigrated prior to reaching older adulthood. Having a better understanding of this distinction can provide more accurate data for health and wellbeing strategies.

Experiences of racial discrimination are known to contribute to health disparities. Systematic reviews and meta-analyses have provided clear evidence that perceived discrimination has a robust relationship with mental and physical health (Lewis et al., 2015; Paradies et al., 2015; Pascoe & Richman, 2009), while a significant association between perceived discrimination and health outcomes has been generally reported among Asian immigrants (e.g., Gee et al., 2009; Mann-Jackson et al., 2018; Williams et al., 2003). Discrimination can deter Asian immigrants from seeking social and health care services (Chen et al., 2020, 2021) and diminish their social and community participation opportunities (Paradies et al., 2015). Since the COVID-19 epidemic, instances of harassment, racial slurs, and hate speech toward Chinese immigrants and other Chinese-looking Asians have substantially increased globally (Jeung & Nham, 2020), contributing to higher depression, anxiety, and reduced life satisfaction (Litam & Oh, 2020).

The impact of the COVID-19 pandemic on older people’s psycho-social wellbeing has been well documented (e.g. Aslan & Onal, 2023; López et al., 2022; Parlapani et al., 2020) and some research has indicated that the additional stressors and psycho-social challenges older people experience because of the pandemic will have ongoing detrimental mental health repercussions (Flint et al., 2020). Increasing discrimination against Asian Americans since March 2020 had resulted in heightened xenophobia (Borja et al., 2020) and intensified concerns for the psychological wellbeing of Chinese immigrants (Jeung et al., 2022). Several international studies have pointed to a significant link between COVID-19 related racism toward Asian populations and negative mental health outcomes, including symptoms of depression, post-traumatic stress, anxiety, and lower levels of life satisfaction (Gao & Liu, 2021; Lee & Waters, 2020; Litam & Oh, 2020; Wong et al., 2020; Woo & Jun, 2021). Furthermore, older immigrants may experience existential threat posed by COVID-19 related to feelings of insecurity about their health and socio-economic wellbeing (Pan et al., 2021).

Compared to other Western countries where increased racist sentiment and comments against Asian communities became apparent during the pandemic (Croucher et al., 2020), the NZ government’s handling of racial relations was deemed more appropriate and sensible, focussing on the importance of solidarity across ethnicities (Liu & Ran, 2020). However, NZ has also experienced this increase in discrimination during the pandemic. The New Zealand Human Rights Commission (2021) reported that 40% of ethnic Chinese respondents had experienced COVID-related discrimination and research has shown that between 23% and 40% of Asian New Zealanders experienced racial discrimination during the pandemic (Jaung et al., 2022; Liu et al., 2022). Apart from Jaung et al.’s finding that experiences of racism were related to lower life satisfaction, there has been little work on the impact of rising discrimination on the health and wellbeing of Chinese immigrants during the pandemic and no investigations of the experiences of older Chinese immigrants.

We aimed to examine the effects of perceived discrimination on depressive symptoms among older Chinese immigrants in NZ during the pandemic. Abbot et al. (2003) and Liu et al. (1997) have already highlighted depression as a focus of concern regarding the mental health of older Chinese immigrants, while perceived discrimination has been linked to symptoms of depression (Pascoe & Richman, 2009).

The evidence for pathways from perceptions of discrimination to depression suggests that loneliness and anxiety mediate the relationship. First, experiences of discrimination are stressors that are related to higher levels of anxiety and a sense of loneliness (e.g., Ormiston et al., 2024). Second, longitudinal research (Cacioppo et al., 2010; Lee et al., 2021) has shown that loneliness predicts depressive symptoms and not the reverse, regardless of other socio-demographic factors. Anxiety symptoms are generally expected to be co-morbid with depressive symptoms with high correlation and co-variance and there is also longitudinal evidence that anxiety predicts depression (Jacobson & Newman, 2014), whereas depression is not accompanied by secondary anxiety (Frances et al., 1992).

Definitive causal models of these relationships remain controversial. Accordingly, we suggest that state anxiety symptoms (in stressful conditions) and loneliness will be related to depressive symptoms and mediate the relationship between perceived discrimination and depression, while not ruling out other directional effects. In addition, our proposed model accounts for the effects of other important stressors identified in the literature: chronic illness (Yeung & Breheny, 2021; Yeung et al., 2020), language barriers (Bhatia et al., 2024), and perceptions of COVID-19 risk (Pan et al., 2021). Demographic variables known to be related to loneliness, anxiety and depression, age, gender, education, marital status, economic wellbeing, and living situation (Aylaz et al., 2012; Curran et al., 2020) were also controlled for in the model.

We hypothesized that:

1. Higher levels of stressors (i.e., perceived discrimination, language barriers, chronic illness, and perceptions of COVID-19 risk) will be related to higher levels of anxiety and loneliness.
2. Anxiety and loneliness will be positively correlated with depression.
3. Anxiety and loneliness will mediate the relationship between perceived discrimination and depression (taking into account language barriers, perceptions of COVID-19 risk and demographic variables).

## Methods

### Study Population and Procedure

A convenience sample of older Chinese immigrants provided cross-sectional data regarding their experiences, needs and wellbeing during the COVID-19 pandemic. Chinese immigrants aged between 55 and 80 who had been living in NZ since the pandemic lockdown (March 2020) were invited to respond to the survey via existing contact databases held by a leading Asian social and community organisation which was the key partner in this research. The Dillman (2007) sample size calculation for population surveys, employing a finite population correction to account for the size of the target population, was used to calculate the target responding sample size. Based on 2013 census data, it was determined that a Chinese immigrant population sample of *n* = 1066 participants would be adequate.

Initially participant recruitment and data collection were planned for mid-year 2021. However, NZ experienced heavy pressure from the Delta variant during 2021, which resulted in another national lockdown lasting around three weeks in August and the region targeted for data collection remained in lockdown until at the end of 2021. While the lockdown was lifted in early 2022, the rapidly growing number of Omicron cases in the community was a concern to the partner organisation and following consultations. It was decided that their team would recruit half of the participants by telephone to distribute and collect the surveys and half would be collected online. The use of telephone and online recruitment methods in health research has been recommended as an alternative during COVID-19 (Amon et al., 2014; Heerman et al., 2017).

The survey was translated into simplified Chinese and distributed to a community sample in April to May 2022 by the partner organisation. Paper surveys were distributed with a reply-paid envelope and an option for contactless pickup by partner organisation team members. The online survey was powered by WeChat to reach out to other potential participants who were not in the organisation’s database. As a token of appreciation, participants who completed the paper-based survey were able to use the identification numbers to redeem a 2-L bottle of cooking oil and those who completed the survey online were sent a code to redeem the oil from the partner organisation. Study procedures were approved by Massey University Human Ethics Committee (SOB 21/26).

A total of 1159 questionnaires was completed, with 54% from paper-based (*n* = 625) and 46% (*n* = 534) from the online portal. A majority were female (61%), 31% were in the age group of 65–69, over 30% had post-secondary school qualifications and most were married (81%). Over 50% had been living in NZ for more than 15 years. Only 11% reported living alone and 40% reported their primary residence was owned by their child. Over 80% were not in paid employment. Of the 1159 participants, 223 (19.2%) expressed feeling discriminated against during the COVID-19 pandemic. To account for missing data for other variables, Little’s Missing Completely at Random test was used, and the result was not statistically significant (*p* = .168), suggesting that data was missing at random. Therefore, mean imputation was employed (Graham, 2009).

### Measures

#### Perceived Discrimination

Perceived discrimination was assessed with 1 = yes/0 = no answers to two questions related to the participants’ experiences since the COVID-19 pandemic: “have you felt discriminated against due to being Chinese?” and “have you reduced interactions or activities due to concerns about discrimination?” These items and scorings came from the New Zealand Human Rights Commission (2021). Scores were recoded as 0 = no discrimination experienced at all and 1 = experienced discrimination from either one of the questions.

#### COVID-19 Risks

*Three questions were developed from the literature review (*e.g.*,* Ro et al., 2021*) to assess* the extent to which COVID-19 was perceived to have negatively affected a participant’s mental health, physical health and economic wellbeing. Response categories included “1 = not at all,” “2 = a little,” “3 = moderately,” “4 = quite a bit,” and “5 = extremely.” Cronbach’s alpha was 0.83. A summed score of the three items was used for analyses.

#### Language Barriers

Four questions were developed based on existing research, particularly from Aotearoa/NZ (Wright-St Clair & Nayar, 2017; Yeung & Allen, 2020; Zhang et al., 2013) to ask participants whether, since the start of the COVID-19 pandemic, they had experienced anxiety, difficulties, delay, and inability to access health or social care services due to language barriers. Scores were recorded as 0 = no and 1 = yes. Cronbach’s alpha was 0.86. A summed score of the four items was used for analyses.

#### Anxiety

Symptoms of anxiety were assessed with the Geriatric Anxiety Inventory short form (GAI-SF: Byrne & Pachana, 2011), designed for the assessment of older adult populations in epidemiological studies. The GAI-SF comprises five questions assessing the presence of symptoms of anxiety in the past seven days against response options coded 0 ‘No’ or 1 ‘Yes’ with a summed total score range 0–5. Established cut off scores were used to describe the prevalence of clinically significant symptoms of anxiety (GAI-SF scores ≥3: Byrne & Pachana, 2011). A validated simplified Chinese translation of this measure (Dow et al., 2018) was administered.

#### Loneliness

*Loneliness* was assessed using the de Jong Gierveld Loneliness Scale (de Jong Gierveld et al., 2009). Participants indicated the degree to which three items reflecting experiences of social loneliness (e.g. ‘there are plenty of people I can rely on when I have problems’) and three items reflecting experiences of emotional loneliness (e.g. ‘I experience a general sense of emptiness’) applied to the way they feel now. Response options were ‘1 = yes’, ‘2 = more or less’, or ‘3 = no’. Items were recoded to ‘0 = no’, ‘1 = more or less’, ‘2 = yes’. The total score ranged from 0 to 6 with scores ≥2 considered to indicate loneliness. A validated simplified Chinese transition of this measure (Leung et al., 2008) was administered to the cohort. Cronbach’s alpha was 0.71.

#### Depressive Symptoms

Depression was assessed using the Centre for Epidemiologic Studies Depression Scale (CESD-10; Björgvinsson et al., 2012; Radloff, 1977). The CESD-10 asks respondents to assess ten depressive symptoms within the last seven days. Each item is rescaled to reflect symptom frequency from ‘0 = rarely or none of the time’ to ‘3 = all the time’. The total score ranges from 0 to 30, with scores >10 indicating significant depressive symptoms. This measure has a high sensitivity (.85) and specificity (.80) to current diagnoses of depression (Boey, 1999). The CESD-10 was validated for use in general populations and indicated adequate reliability and validity for the community-dwelling older population in China (Chen & Mui, 2014). Cronbach’s alpha was 0.79.

#### Chronic Illness

To assess chronic health conditions participants were asked to indicate whether a health professional had ever diagnosed them with a list of seven specified chronic health conditions (e.g. chronic kidney/renal disease, diabetes). The number of those ticked as ‘yes’ was reported as a summed score labelled ‘total health conditions’ (range 0–7).

#### Demographic Variables

Participants were asked to report their age (young: 55–74; old: 75–80+), gender (male, female or gender diverse), education qualification (no qualifications, secondary school, post-secondary certificate, diploma, or trade diploma), marital status (married/partnered; other), living arrangement (alone; with others), employment status (paid employment, not in paid work or other), housing tenure (own by themselves or other), and year of first immigration to NZ.

Economic wellbeing was assessed using one of the items from the Economic Living Standards Index short form (ELSI-SF; Jensen et al., 2005). The item asked participants to assess ‘How well does your total income meet your everyday needs for such things as accommodation, food, clothing, and other necessities?’ ranging from 1 = not enough to 4 = more than enough.

### Statistical Analyses

The IBM SPSS Statistical package (version 28, IBM SPSS Statistics for Windows) was used for data entry and analysis. Preliminary assumption testing was first conducted to check for homogeneity of variance across groups (Tabachnick & Fidel, 2019). This assumption was not violated.

Analyses were conducted in four phases. In phase one, Crosstabs with chi-square (χ^2^) were employed to compare Chinese older immigrants who experienced discrimination with those who did not on all categorical factors (e.g. gender and educational level) and independent *t*-tests were used to assess differences between the groups on wellbeing factors (e.g. health conditions and loneliness).

In phase two we entered all proposed measures into a correlation matrix to identify significant bivariate relationships and to check that the independent variables were at least minimally (≥0.20) correlated with depression and that no independent variables were too highly correlated (≥0.80) with one another. Two-tailed tests were utilised (*p* < .05) to identify significant associations.

In phase three, a hierarchical regression was performed to evaluate the associations between independent variables (perceived discrimination, numbers of chronic illnesses, language barriers, and COVID-19 risks), anxiety and loneliness and depression. Socio-demographic variables (i.e. age, gender, education, marital status, and living situation) were controlled in all regression calculations. Lastly, the mediation effect of anxiety and loneliness was tested using the SPSS macro PROCESS using model 6. PROCESS macro provides the mechanism to estimate direct and indirect effects in single and multiple mediator models. Mediation analyses were performed for the evaluation of the following effects: (1) the independent variables (perceived discrimination, numbers of chronic illnesses, language barriers and COVID-19 risks) on the dependent variable (depression); (2) the independent variable on each mediator (anxiety and loneliness); and (3) the independent variable and the potential mediators on the dependent variable. Bootstrapping was applied to empirically estimate the sampling distribution of the indirect effect and generate a bootstrapping confidence interval (95% CI) based on 5000 bootstrap samples for bias corrected bootstrap CIs (Hayes, 2017). If the 95% CI of the mediation effect did not contain zero, then the effect would be significant at the 0.05 level.

As repeated measures across the groups could lead to an increased chance of making a Type I error, a Bonferroni adjustment was made to the alpha level to reduce that possibility as recommended (Tabachnick & Fidel, 2019). The minimal level of significance was set at *p* < .005 (i.e. 0.05/11 = 0.005) for these comparisons.

## Results

Participants who completed the paper-based survey were compared to those who completed via WeChat. Although there were no significant differences among socio-demographic indicators (i.e. gender, marital status, living arrangement, employment status, and housing tenure) between the two groups, descriptive results indicated Chinese immigrants who were older with lower educational qualifications chose to use the paper-based method. Those who completed the paper-based format also reported significantly poorer health (*p* = .001) and poorer economic wellbeing (*p* = .001).

Table 1 indicates that there were no significant differences between the discrimination/no-discrimination groups on socio-demographic indicators (i.e. age group, gender, education level, marital status, living arrangement, and time living in Aotearoa NZ). However, those who experienced discrimination reported significantly more health conditions, lower economic wellbeing, higher perceived COVID-19 effects on their health and financial status, more delay, and difficulties in accessing social and health care services due to language barriers, greater loneliness, higher levels of anxiety and depression, than those not experiencing discrimination (see Table 2). According to the cut-off point for anxiety (≥3) and depression (CESD-10 ≥ 10), 45% of the participants who felt discriminated against indicated clinically significant anxiety symptoms, and 58% reported significant depressive symptoms compared to those who did not experience discrimination during the outbreak of COVID-19.

**Table 1.** Comparison of Sociodemographic Among Older Chinese Immigrants Who Experienced Discrimination During the COVID-19 Pandemic.

	Total (*N* = 1159)	Felt discriminated (*N* = 223)	Not at all (*N* = 919)	Statistics	*p*-value	95%CI
Variables						
Age				*χ*^2^ = 3.45	---	--
55–59	97 (8.4)	25 (11.3)	71 (7.8)			
60–64	163 (14.1)	32 (14.4)	131 (14.4)			
65–69	355 (30.6)	70 (31.5)	283 (31.2)			
70–74	259 (22.3)	46 (20.7)	211 (23.3)			
75–79	167 (14.4)	32 (14.4)	128 (14.1)			
80+	103 (8.9)	17 (7.7)	83 (9.2)			
Gender				*χ*^2^ = .51	---	--
Male	442 (38.1)	86 (39.3)	350 (38.6)			
Female	698 (60.2)	133 (60.7)	555 (61.2)			
Gender diverse	2 (0.2)	0 (0.0)	2 (0.2)			
Educational level				*χ*^2^ = 3.63	---	--
None	121 (10.4)	30 (13.7)	87 (9.7)			
Secondary school	321 (27.7)	64 (29.2)	253 (28.1)			
Post-secondary	368 (31.8)	68 (31.1)	298 (33.1)			
University degree	323 (27.9)	57 (26.0)	262 (29.1)			
Marital status						
Married/Partnered	1017 (87.7)	191 (89.3)	810 (89.5)	*χ*^2^ = .01	---	--
Other	118 (10.2)	23 (10.7)	95 (10.5)			
Living arrangement						
Alone	124 (10.7)	28 (12.6)	93 (10.1)	*χ*^2^ = 1.12	---	--
With others	1035 (89.3)	195 (87.4)	826 (89.9)			
Time living in aotearoa NZ (years)	16.3 (9.4)	16.7 (9.1)	16.1 (9.6)	*t* = −0.79	*---*	--

*p < .05, **p < .005*;* CI = confidence interval.

**Table 2.** Comparison of Psychosocial, Health and Wellbeing Indicators Among Older Chinese Immigrants Who Experienced Discrimination During the COVID-19 Pandemic.

	Felt discriminated (*N* = 223)	Not at all (*N* = 919)	Statistics	*p*-value	95%CI	Effect size ‘d' or ‘φ’
Variables						
Total health conditions	1.3 (1.5)	1.1 (1.34)	*t* = −2.6	<.005**	−.35–−.06	.20
Income meeting needs	1.8 (0.6)	2.0 (0.7)	*t* = 4.3	<.005**	.11–.29	.31
COVID-19 impact on physical health	2.7 (1.2)	2.3 (1.1)	*t* = −4.9	<.005**	−.60–−.26	.35
COVID-19 impact on mental health	3.0 (1.1)	2.4 (1.1)	*t* = -6.3	<.005**	−.67–−.35	.55
COVID-19 impact on economic wellbeing	3.0 (1.1)	2.5 (1.1)	*t* = -5.3	<.005**	−.60–−.28	.46
COVID-19 risks - total	8.5 (2.9)	7.2 (2.9)	*t* = -6.3	<.005**	−1.77–−.93	.45
Language barrier – experienced anxiety to access health/social services during COVID-19			*χ*^2^ = 111.9	<.005**	.26–.38	.32
Yes	155 (70.8)	290 (31.9)				
No	64 (29.2)	620 (68.1)				
Language barrier – delayed obtaining health/social services during COVID-19			*χ*^2^ = 56.8	<.005**	.16–.29	.23
Yes	106 (50.5)	218 (24.2)				
No	104 (49.5)	682 (75.8)				
Language barrier – experienced difficulties in accessing health/social services during COVID-19			*χ*^2^ = 62.0	<.005**	.18–.30	.24
Yes	108 (51.4)	216 (24.0)				
No	102 (48.6)	684 (76.0)				
Language barrier – unable to access health/social services during COVID-10			*χ*^2^ = 45.7	<.005**	.14–.28	.20
Yes	81 (38.6)	156 (17.3)				
No	129 (61.4)	744 (82.7)				
Language barriers - total	2.01 (1.5)	1.0 (1.4)	*t* = -9.9	<.005**	−1.34–−.91	.67
Anxiety	2.3 (2.0)	1.2 (1.8)	*t* = −7.1	<.005**	−1.35–−.77	.58
No anxiety symptom	122 (54.7)	697 (75.8)	*χ*^2^ = 39.5	<.005**	.13–.25	.19
Have anxiety symptom	101 (45.3)	222 (24.2)				
Loneliness	4.1 (1.6)	3.1 (1.8)	*t* = −8.2	<.005**	−1.24–−.76	.59
Depression	11.3 (6.0)	8.0 (5.7)	*t* = −7.5	<.005**	−4.10–−2.40	.56
No depressive symptom	89 (42.2)	566 (63.8)	*χ*^2^ = 33.1	<.005**	.12–.24	.17
Have depressive symptom	122 (57.8)	321 (36.2)				

*p < .05; **p < .005; CI = confidence interval; ‘d’: small = 0.2, medium = 0.5, large = 0.8; **‘φ’**: small = 0.1 to 0.29; medium = 0.30 to 0.49, large ≥0.50.

Table 3 indicates that depression was positively associated with all COVID-19 risks, and anxiety and loneliness, (≥0.20) which were accordingly included in the regression analysis. Table 4 presents the results of the hierarchical multiple regression. Step 1 including the demographic variables accounted for 4% of the variance in depression scores with age, gender, education, and living situation making a significant contribution. When discrimination, chronic illnesses, language barriers, and COVID-19 risk were entered at Step 2, the variance increased to 31% (an increment of 27%) and all variables contributed significantly. At Step 3, both anxiety and depression significantly added an additional 20%, resulting in a total 51% variance in depression scores explained by age, language barriers, COVID-19 risks, anxiety, and loneliness. In order of the size of the unique prediction of variance in depression scores were: anxiety (β = 0.40, <0.005); loneliness (β = 0.23, <0.005); COVID-19 risks (β = 0.19, <0.005); age (β = 0.09, <0.005), and language barriers (β = 0.07, <0.005). Based on PROCESS (see Table 5), anxiety and loneliness were shown to be significant mediators between the independent variables and depression.

**Table 3.** Correlation Matrix of Self-Rated Health and Independent Predictors.

Variables	1	2	3	4	5	6
1. Depression	--					
2. Discrimination	0.22**	--				
3. Anxiety	0.60**	0.22**	--			
4. Loneliness	0.47**	0.22**	0.37**	--		
5. COVID risks	0.43**	0.18**	0.34**	0.26**	--	
6. Language barriers	0.30**	0.23**	0.27**	0.22**	0.29**	--
7. Chronic illnesses	0.23**	0.08**	0.25**	0.17**	0.17**	0.29**

*p < .05, **p < .005.

Effect size: Cohen *r* of 0.3 = medium; *r* of 0.5 = large.

**Table 4.** Regression Model of Depression on Other Independent Variables Among Older Chinese Immigrants.

Variables entered	*Step 1*			*Step 2*			*Step 3*		
	*B*	*Beta (95% CI)*	*p-value*	*B*	*Beta (95% CI)*	*p-value*	*B*	*Beta (95% CI)*	*p-value*
Age	0.31	0.07 (0.05–0.56)	0.02*	0.36	0.08 (0.14–0.58)	0.02*	0.40	0.09 (0.21–0.59)	<0.005**
Gender	0.91	0.08 (0.19–1.64)	0.01*	1.02	0.09 (0.40–1.64)	0.01*	0.69	0.06 (0.17–1.22)	0.01*
Education	−0.63	−0.10 (−1.0–−0.27)	<0.005**	−0.32	−0.05 (−0.63–−0.01)	0.04*	−0.29	−0.05 (−0.55–−0.03)	0.03*
Marital status	−1.25	−0.07 (−2.50–0.00)	0.05	−1.30	−0.07 (−2.36–−0.23)	0.02*	−0.75	−0.04 (−1.65–0.15)	0.10
Living situation	1.56	0.08 (0.14–0.58)	0.01*	1.08	0.06 (0.02–2.15)	0.05	0.35	0.02 (−0.55–1.25)	0.45
Discrimination		--		1.27	0.12 (0.71–1.82)	<0.005**	0.42	0.04 (−0.06–0.89)	0.09
Chronic illnesses		--		0.60	0.14 (0.38–0.82)	<0.005**	0.19	0.04 (0.00–0.38)	0.05
Language barriers		--		0.68	0.17 (0.47–0.90)	<0.005**	0.27	0.07 (0.09–0.46)	<0.005**
COVID-19 risks		--		0.67	0.33 (0.56–0.78)	<0.005**	0.39	0.19 (0.29–0.48)	<0.005**
Anxiety					--		1.29	0.40 (1.13–1.45)	<0.005**
Loneliness					--		0.75	0.23 (0.59–0.90)	<0.005**
*R, R* ^*2*^	0.21, 0.04			0.55, 0.31			0.71, 0.51		
*∆R* ^*2*^	0.04, *F* (5, 1085) = 10.08			0.30, *F* (4, 1081) = 101.637			0.50, *F* (2, 1079) = 222.79		

*p < .05; **p < .005 CI = confidence interval.

Gender (1 = male, 0 = female and others); Education (1 = secondary or higher, 0 = no qualifications); Marital status (1 = married/partnered, 0 = other); Living situation (1 = living alone, 0 = living with others).

**Table 5.** Indirect Effects on Depression Among Chinese Older Immigrants via Anxiety and Loneliness.

Path (Indirect effect of chronic illnesses, discrimination, COVID-19 risks and language barriers on depression	Coefficient^a^	LL	UL
Chronic illnesses → anxiety → depression	0.12	0.09	0.15
Chronic illnesses → loneliness → depression	0.02	0.01	0.04
Chronic illnesses → anxiety → loneliness → depression	0.03	0.02	0.03
Total indirect effects (standardised)	0.17	0.13	0.21
Total effect (unstandardised)	0.99	0.75	1.23
Discrimination → anxiety → depression	0.11	0.08	0.15
Discrimination → loneliness → depression	0.04	0.03	0.06
Discrimination → anxiety → loneliness → depression	0.02	0.01	0.03
Total indirect effects (standardised)	0.18	0.14	0.22
Total effect (unstandardised)	2.60	2.00	3.19
COVID-19 risks → anxiety → depression	0.15	0.12	0.18
COVID-19 risks → loneliness → depression	0.03	0.02	0.05
COVID-19 risks → anxiety → loneliness → depression	0.03	0.02	0.04
Total indirect effects (standardised)	0.22	0.18	0.25
Total effect (unstandardised)	0.88	0.78	0.99
Language barriers → anxiety → depression	0.13	0.10	0.16
Language barriers → loneliness → depression	0.03	0.02	0.05
Language barriers → anxiety → loneliness → depression	0.02	0.02	0.03
Total indirect effects (standardised)	0.19	0.15	0.22
Total effect (unstandardised)	2.24	1.82	2.65

^a^completely standardised effect; *LL* lower limit; *UL* Upper limit (95% Confidence Interval).

## Discussion

This study aimed to examine whether perceived discrimination (and number of chronic illnesses, language barriers, and COVID-19 risk perceptions) would predict depression, via anxiety and loneliness as mediators, among older Chinese immigrants. All three hypotheses were supported. The regression model supported the prediction that discrimination, chronic health conditions, language barriers, and perceived COVID-19 risks were indirectly related to depression through higher anxiety and loneliness. There were also direct effects of language barriers and perceptions of COVID-19 risks on depression. These findings extend understandings of the link between discriminatory experiences and mental health, as well as the important roles of inequalities due to language barriers among older Chinese immigrants living in NZ during the COVID-19 pandemic.

Nearly one fifth of the sample of older Chinese immigrants reported experiences of or fear of discrimination during the pandemic. Others have reported higher levels of discrimination (Jaung et al., 2022; Liu et al., 2022); however, these were predominantly reported by younger people, students, temporary visa holders, or rural area participants. Our results suggest that older Chinese people living in Auckland, NZ were less likely to report discrimination than these wider groups. Regardless, the present findings highlight experiences of discrimination as an important mental health risk for older Chinese immigrants, consistent with existing research on Chinese immigrants (Yang et al., 2022).

Support for our mediation model, based on understandings of discrimination as a stressor resulting in increased anxiety and loneliness, highlights pathways through which discrimination may affect depression. The finding that loneliness and anxiety fully mediate the relationship between perceived discrimination and depressive symptoms adds further theoretical explanation to international empirical reports during the pandemic. While perceived discrimination has been shown to be related to depression and anxiety (Yu et al., 2022) or psychological distress and loneliness (Lee et al., 2021; Yang et al., 2022) among Chinese immigrants, our findings provide additional information about the direction of the relationships among these variables. These results support the suggestion of Lee and Bierman (2019) that loneliness is a mediator between discrimination and depression and additionally suggest the importance of anxiety as a pathway. Further research into these explanatory pathways is encouraged to provide additional support for the model.

Older Chinese immigrants may have heightened vulnerability to the health effects of discrimination. Our model also included some of the broader challenges faced by older Chinese immigrants. Chronic health conditions (Yeung et al., 2020) and language barriers (a recognised difficulty for older immigrants; e.g. Bhatia et al., 2024) were also stressors that contributed to the model and were directly related to anxiety, loneliness, and in the case of language barriers, to depression. The interrelationships among these variables, including relationships with discrimination, point to a complex of stressors that must be taken into account when considering interventions with individuals and communities.

The strongest relationship with all aspects of mental health in the model was shown for the perceptions of physical, mental and economic risks from COVID-19. This finding highlights the context of the study. A stressful situation in itself, the pandemic exacerbated reports of discrimination, and perceptions of personal risk explained additional anxiety, loneliness and depression among the sample. This heightened level of stress, and related mental health issues, may not be the norm, but does provide a sharpened picture of the wider stressors faced by older Chinese immigrants in any context. In terms of pandemic situations, the findings reveal important aspects of vulnerability and focus for intervention and support and contribute to the literature focussing on the support needs of older Chinese immigrants (e.g. Bhatia et al., 2024; Park & Kim, 2013; Wang et al., 2020; Zhang et al., 2013; Zhao et al., 2022).

Racial discrimination itself cannot be counteracted through individual support. A recent scoping review on a decade of Asian and ethnic minority health research in Aotearoa NZ revealed the limited published studies in quantity and the research areas covered (Chiang et al., 2021). This may reflect the ongoing lack of funding to support Asian health research and systemic racism relating to the invisibility of the discourse on Asian health in Aotearoa NZ’s health agenda and policies (Wong, 2015). There have also been suggestions for moves to counteract discriminatory behaviour and its effects in the wider society. Takamura et al. (2022) suggested several policy mechanisms to combat hate and violence against older Asian Americans, that have resonances for many Western countries. These authors have suggested some of the guidelines and frameworks such as the expansion of civil rights protection to include protection for older persons in public community settings, with emphasis on those at risk because of race or ethnicity; government initiatives to advance equity, justice, and opportunities for participation including public safety; the collection of reliable disaggregated and intersectional data; and education and public information to increase understanding of discrimination at all levels. Wang et al. (2020), when considering the experiences of older Chinese in Canada, also suggested that government agencies should develop anti-racism policies and practices. In regard to practices, Wang et al. also suggested that social workers and other gerontological practitioners should attend to the strengths that cross-national identities provide and assist older people to draw on these strengths. One mechanism suggested is facilitation of the uptake of digital communication to enable older people to continue to be engaged in society.

### Limitations and Strengths

A strength of this study is the inclusion of personal and contextual factors in the study of discrimination, and the consideration of the effects of other stressors including the context of a pandemic. However, a limitation was the cross-sectional nature of the analyses and the direction of the relationships. For example, it is likely that depression could be affecting people’s anxiety. The theoretical model tested here was concerned with highlighting the pathways that explain the known relationship of discrimination to depression. The role of the model that we tested is to introduce the important cross-sectional relationships among these variables, rather than rigorously test the direction of those relationships. Although the general set of relationships proposed is supported, longitudinal analyses could be used to further delineate the direction of these relationships.

Another limitation is that the total variance of the model to depression was 51%, suggesting that the depressive symptoms experienced during COVID-19 by older Chinese immigrants were not fully explained by the predictors in this model. Future studies will need to consider including measures that may capture the cultural nuances of Chinese immigrants’ values and perceptions and other factors that might predict risk perceptions and self-rated health for model testing.

Perceived discrimination was an important factor in this study. However, the use of a dichotomous variable limited the explanatory power of the analysis. The variables were included in a space limited questionnaire developed at the time of pandemic, and as such were unable to capture important nuances such as frequency, intensity, or type. Future research would make a valuable contribution by developing improved measures of perceived discrimination.

The study used two different modes of data collection. Despite the known merits of telephone and online recruitment methods, there are potential limitations such as lack of diversity in age and/or socioeconomic status (Villarosa et al., 2021). While there were no differences between those who completed the paper-based questionnaire and those who completed the survey on-line according to gender or marital status, there were socio-economic and educational differences. Those who completed the paper-based survey were more likely to be older with lower education qualifications, poorer health, and poorer economic wellbeing. In this situation, two methods of data collection were a strength enabling the recruitment of a broader range of people from different circumstances.

## Conclusion

This study provided critical information on perceived discrimination as a significant risk factor for higher levels of depression among older Chinese immigrants in NZ. The findings contribute to international evidence for the effects of language barriers, chronic illnesses, COVID-19 risks on mental, physical, and financial wellbeing, and perceived discrimination in the context of a pandemic among older Chinese immigrants. Given the social and health inequalities among racialized groups and individuals in NZ (e.g. Montayre et al., 2019; Stephens et al., 2022; Szabo, 2022) and the rising cases of racism and discrimination against Asian people living in NZ since the start of the COVID-19 outbreak (Liu et al., 2022), as well as the more prolonged mental health impacts of the pandemic on older populations (Aslan & Onal, 2023), older Chinese immigrants are the focus of growing concern for mental health issues during and following the pandemic. While older Chinese immigrants have been continuously underserved in mental health support and services due to cultural, language, and social barriers, this study also adds to a growing literature in this area to support a link between recent discriminatory experiences and their impact on health and wellbeing among older Chinese immigrants. Suggestions for anti-racist policies and practices by social workers and other professionals that support Chinese immigrants in the face of racism and ageism in host countries should be implemented.
